# Epidemiology of *Strongyloides stercoralis* infection in Bolivian patients at high risk of complications

**DOI:** 10.1371/journal.pntd.0007028

**Published:** 2019-01-17

**Authors:** Laurent Gétaz, Rosario Castro, Pablo Zamora, Marcelo Kramer, Nestor Gareca, Maria del Carmen Torrico-Espinoza, José Macias, Susana Lisarazu-Velásquez, Gloria Rodriguez, Carola Valencia-Rivero, Thomas Perneger, François Chappuis

**Affiliations:** 1 Division of Tropical and Humanitarian Medicine, Geneva University Hospitals and University of Geneva, Geneva, Switzerland; 2 Division of Prison Health, Geneva University Hospitals and University of Geneva, Geneva, Switzerland; 3 Division of Infectious Diseases, Hospital Clínico VIEDMA, Cochabamba, Bolivia; 4 Division of Parasitology, Centro Nacional de Enfermedades Tropicales (CENETROP), Santa Cruz, Bolivia; 5 Center for Rheumatic Diseases M. Kramer, Santa Cruz, Bolivia; 6 Division of Rheumatology, Hospital Clínico VIEDMA, Cochabamba, Bolivia; 7 Division of Hematology & Oncology, Hospital Clínico VIEDMA, Cochabamba, Bolivia; 8 Division of Hematology & Oncology, Clinica Los Olivos, Cochabamba, Bolivia; 9 Centro Departamental de Vigilancia y Referencia de Enfermedades de Transmisión Sexual ITS/VIH/SIDA (CDVIR), Santa Cruz, Bolivia; 10 Instituto Oncológico del Oriente Boliviano (IOOB), Santa Cruz, Bolivia; 11 Programa Nacional ITS/VIH/SIDA/VH, Ministerio de Salud, La Paz, Bolivia; 12 Division of Clinical Epidemiology, Geneva University Hospitals, and Faculty of Medicine, University of Geneva, Switzerland; George Washington University, UNITED STATES

## Abstract

**Background:**

Strongyloidiasis can be fatal in immunocompromised patients, but few epidemiological studies investigated the burden of this neglected tropical disease among these populations, particularly in low- and middle-income countries such as Bolivia. This study aimed to fill in this gap by estimating prevalence rate and risk factors associated with strongyloidiasis among patients at high risk of complications

**Methods:**

A cross-sectional study was carried out in Santa Cruz (elevation 400 meters, tropical climate) and Cochabamba (elevation 2,500 meters, temperate climate), among patients with cancer, HIV infection and rheumatic or hematologic disease, using four coproparasitological techniques and one serological (ELISA) test.

**Results:**

In total, 1,151 patients participated in this study, including individuals who were HIV-positive (30%) or with rheumatic (29%), oncologic (32%) or hematologic (9%) diseases. The serological and coproparasitological prevalence was 23.0% (95% confidence interval [CI], 20.7–25.5; n = 265/1151) and 7.6% (95% CI, 6.2–9.3; n = 88/1151), respectively, with an estimated actual prevalence of 20.2% (95% CI, 17.9–22.5). Positive serology and positive coproparasitology were associated with younger age and lower education levels. There was no significant difference in prevalence between Cochabamba and Santa Cruz as defined by coproparasitology (6.4% *vs*. 8.9%; p = 0.11) or serology (24.0% *vs*. 22.0%; p = 0.4). Among 64 patients in Cochabamba who had never travelled to the tropical lowlands, 5 (7.8%) had a positive coproparasitology.

**Conclusions:**

Strongyloidiasis is widely prevalent in Bolivia among vulnerable patients at increased risk of life-threatening complications. Transmission of the parasite occurs both in tropical lowlands and temperate elevation (≥ 2,500 m). Control strategies to prevent transmission and complications of this serious parasitic disease should be urgently reinforced.

## Author summary

Strongyloidiasis is an understudied chronic intestinal infection and one of the most overlooked of the neglected tropical diseases, particularly in countries such as Bolivia. The strongyloidiasis usually remains paucisymptomatic for decades in immunocompetent persons, but may lead to severe conditions with high mortality rates in immunocompromised individuals. We carried out a study to explore the epidemiology of strongyloidiasis among Bolivian patients with cancer, HIV infection, and rheumatic or hematologic diseases. Our study highlighted that strongyloidiasis was widely prevalent in tropical and inter-Andean temperate areas of Bolivia among patients at high risk of life-threatening complications. One in five participants was infected. We showed that strongyloidiasis was highly prevalent in both tropical and temperate regions. Improved access to adequate diagnosis, treatment, and preventive measures are therefore urgently required in Bolivia, and most likely elsewhere in the region. Treatment of uncomplicated strongyloidiasis is simple, highly effective, and potentially life-saving.

## Introduction

*Strongyloides stercoralis* is a soil-transmitted intestinal nematode that causes one of the most overlooked of the neglected tropical diseases [1]. An estimated 370 million individuals (5.2%) are infected with strongyloidiasis worldwide [2]. Moreover this estimation could be underestimated because of the low sensitivity of the usual diagnostic method (i.e., conventional fecal-based analysis for *S*. *stercoralis* diagnosis [3, 4]. The disease is endemic in areas with warm and humid climates, but epidemiological data are largely lacking in low- and middle-income countries such as Bolivia [5].

Typically, strongyloidiasis is contracted by skin penetration of filariform larvae from contaminated soil. Larvae migrate to the lungs before settling in the intestine, where they develop into adult worms. Eggs produced by adult females hatch in the intestine and larvae are released in the stool, while some new-born larvae can penetrate the bowel or the perianal skin. This process of replication by autoinfection leads to chronic infection that can last for decade [6].

Most chronic infections are asymptomatic or give rise to intermittent, mild gastrointestinal, pulmonary or cutaneous symptoms. In immunocompromised patients however, in particular where Th2 responses are weakened, a potentially fatal accelerated autoinfection syndrome may develop that leads to systemic multi-organ complications. The hyperinfection syndrome (HS) involves gastrointestinal and pulmonary systems, and disseminated strongyloidiasis (DS) involves also other organs (*e*.*g*. the liver and central nervous system). Both are often accompanied by life-threatening sepsis involving Gram-negative bacteria following larval penetration of the intestine wall [7, 8].

The prescription of corticosteroids is the most common cause of immunosuppression that may result in HS/DS and sepsis. Corticoids not only reduce Th2 and eosinophilic responses, but moreover increase *Strongyloides* egg production [8]. Several other immunosuppressive conditions predispose to HS/DS-associated complications. These include chemotherapy for cancer, hematological malignancies, HIV-1, human T-cell lymphotropic virus type 1 infection, diabetes mellitus, organ transplantation, alcohol abuse and severe malnutrition. HIV infection predispose to HS/DS under certain conditions (e.g., immune reconstitution syndrome or opportunistic infections requiring corticoid prescription such as *Pneumocystis jirovecii* pneumonia) [3, 9–11].

Treatment of uncomplicated strongyloidiasis is simple and highly effective, and potentially life-saving before immunosuppressive prescription [6]. Severe strongyloidiasis however does not always respond to treatment and mortality rates of up to 87% have been reported in HS/DS [8].

The aim of the study was to estimate the prevalence of and factors associated with strongyloidiasis among patients at high risk of complications living in two Bolivian regions, each with a distinct climate.

## Methods

### Study setting

A cross-sectional study was conducted from July 2012 to April 2013 in nine medical facilities of two Bolivian cities, Cochabamba and Santa Cruz: the public hospital of oncology “Instituto oncológico del Oriente Boliviano” (IOOB), the HIV/AIDS public center “Centro Departamental de Vigilancia y Referencia de ITS/Sida” (CDVIR) and the Center for Rheumatic Diseases Kramer in Santa Cruz, the HIV/AIDS, rheumatology, hematology and oncology units of the public hospital “VIEDMA”, the National Institute of Oncology of Tiquipaya and the Clinic “Los Olivos” in Cochabamba. Cochabamba is located at an elevation of 2,500 meters in an inter-Andean valley, and enjoys a temperate and semi-arid climate. Santa Cruz lies at an elevation of 400 meters and its climate is tropical.

### Study population, inclusion/exclusion criteria and sample size

Inclusion criteria were as follows: all patients who consult in one of the 9 recruitment centers, men or women older than 18 years; suffering from pathologies predisposing to HS/DS: a neoplasm (hematological or solid-organ malignancies), an inflammatory rheumatic disease or HIV infection. Exclusion criteria were: antiparasitic treatment in the past six months, a clinical condition impairing short-term survival, or inability to provide consent. We aimed to recruit 1,200 participants, divided evenly between the two cities and the groups of pathologies. We assumed a prevalence of 16%, and aimed to obtain a 95% confidence interval on the prevalence of ±3% in each city. Patients with hematological pathologies were however under-represented for two reasons: the prevalence of hematological diseases is relatively low compared to the other targeted conditions, and hematologists in Santa Cruz often de-worm their patients.

### Laboratory investigation procedures

#### Stool collection and examination

Clean plastic sheets and wooden applicator sticks were distributed and the participants were instructed to bring two stool specimens to the laboratory at intervals of at least three days. The stool tests were processed within four hours after emission in one of the three reference laboratories of parasitology (VIEDMA, IOOB, CENETROP). Samples were examined by two senior laboratory technicians. A laboratory technician at each site was trained to standardized stool techniques during one week in the Institute of Tropical Medicine “Cayetano Heredia” in Lima-Peru. The technicians were blinded to serological results. Stool samples were analyzed by four methods: *Direct smear examination* (DSE), *formalin-gasoline sedimentation technique* (FGST), a*gar plate culture (APC)* incubated at 32° for four days and *modified Baermann technique* (MBT) as described elsewhere [12–15]. For each of the two stool samples all four coproparasitological diagnostic tests were performed (8 tests per person in total). For each method, positive samples were confirmed at 400x magnification according to caudal pole shape of the parasite, buccal cavity size and structure and localization of the genital primordium. “Positive coproparasitology” is defined by the detection of *S*. *stercoralis* larvae by at least one of the techniques. Fecal based methods are virtually 100% specific, but the diagnosis is hampered by their suboptimal sensitivity, including for Baermann and Agar plate culture, two of the most sensitive methods for the detection of *S*. *stercoralis* in stool [16].

#### Strongyloides ELISA serology

Sera were separated from freshly collected blood and stored at -20° prior to testing. IgG against *Strongyloides ratti* antigen was detected by ELISA as recommended by the manufacturer (Bordier Affinity Products).

### Data collection

Epidemiological, socio-demographic and clinical variables were collected using structured questionnaires, including gender, age, education level, living area (rural/urban) and history of travel to tropical or subtropical areas (for people living in Cochabamba). Other data collected were the detailed status of the infectious (HIV), rheumatic, oncologic or hematologic diseases, history of abdominal pain and diarrhea during the last month, history of skin eruption compatible with larva currens during the last three months, history of immunosuppressive drugs during the last three months (specifically glucocorticoids, methotrexate, cyclophosphamide and azathioprine), quantification of CD4^+^ lymphocytes (for all HIV patients), frequency of barefoot walking outside, and alcohol consumption. The data were compiled into an Excel spreadsheet (see **S1 Dataset**).

### Statistical analyses

Only participants with complete records (two stool samples analyzed, serology done and completed questionnaires) were included in the final analysis.

#### Estimation of the actual prevalence

Using a robust methodology, Bisoffi et al. have assessed the sensitivity (91%) and the specificity (94%) of the serologic test Bordier-ELISA for the detection of *S*. *stercoralis*, among a composite population including also subjects from tropical areas and co-infected with other parasitic infections [16]. We calculated the actual prevalence using the following formula: AP = (OP + Sp – 1) / (Se + Sp – 1), where AP is the actual prevalence, OP is the observed prevalence of positive serological test results found in our study and Sp (94%) and Se (91%) are the specificity and sensitivity estimates found in the study cited earlier [16]. 95% lower and upper confidence limit of the actual prevalence were also calculated AP±1.96xsqrt(AP*(1-AP)/n), where n is the sample size.

#### Associations with risk factors

Bivariate and multivariate logistic regression analyses were carried out to associate potential factors with positive serology and stool examination. Participants with missing data were not included in the related analyses. We decided to include the same independent variables in the multivariate logistic regression models for positive coproparasitology and for positive serology, to facilitate direct comparisons. In these models, we included risk factors that were significantly associated (p<0.05) in bivariate analysis with either outcome (positive coproparasitology or positive serology). In addition, we forced the study site (Cochabamba vs Santa Cruz) into the models, because we believed that the baseline risk may be different, a priori. We did not include abdominal symptoms (diarrhea and pain) as these are likely consequences of parasitic infection, not its causes. Similarly, we did not include coinfections with other intestinal parasites because their presence might reflect shared risk factors with strongyloidiasis, and this would cause overadjustment. Once the models were obtained, we did not exclude non-significant independent variables or attempt to build parsimonious models. P-values <0.05 were considered significant. Statistical analysis was carried out using SPSS 22.0 for Windows (SPPS Inc., Chicago, USA).

#### Performance comparison of coproparasitological techniques

We estimated the sensitivity of each coproparasitological technique among people with *S*. *stercoralis* larvae detected with at least one fecal technique, because the global sensitivity of each test is not computable due to the lack of a gold standard for the diagnosis of *S*. *stercoralis*. For this analysis, stool results of participants processed in the CENETROP laboratory were not included (347 participants, 16 with *S*. *stercoralis* detected in stools). In this laboratory, the performance of DSE and FGST may be overestimated. Indeed, when the MBT was positive, laboratory technicians sometimes spent additional time on the DSE and FGST blades. Nevertheless, this limitation did not affect the overall coproparasitological prevalence.

### Ethical considerations

All patients were adults (age range: 18–84 years old) and were personally informed about the purpose of the study and provided written consent for participation. The study protocol was approved by the Ethics Committee of the Medical College (Santa Cruz), the Ethics Committee of the University Mayor of San Simon (Cochabamba) and the Research Ethics Committee of the Geneva University Hospitals (Switzerland; CER 11–171). Participants who were found positive for parasitic infection (coproparasitological and/or serological tests) were treated free of charge with standard of care, including two doses of ivermectin (200 μg/kg) for *S*. *stercoralis*.

## Results

### Patient characteristics

**Fig 1:**
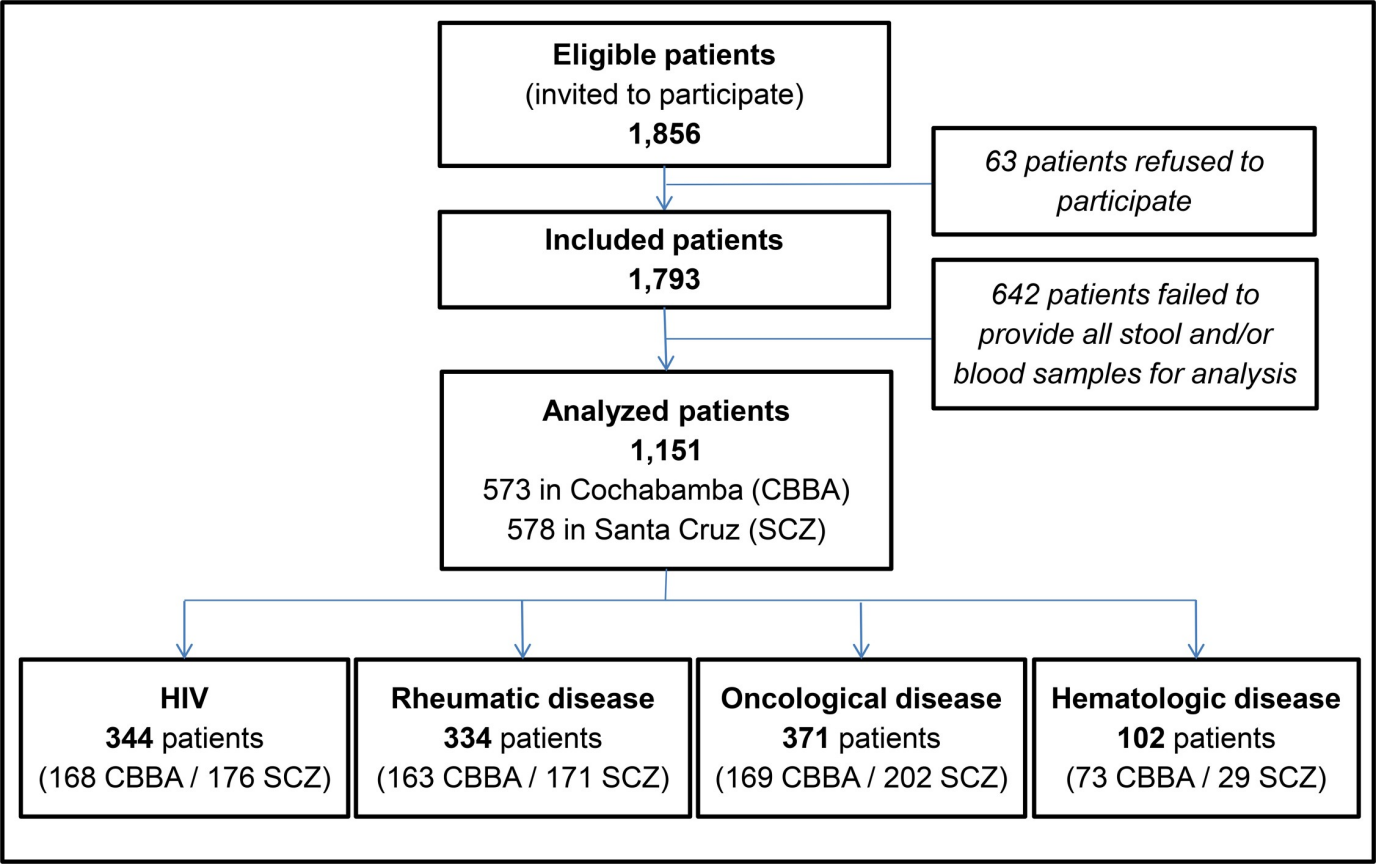
Study diagram with patient disposition.

Among 1,856 patients invited to participate in the study and matching the inclusion and exclusion criteria, 1,151 were included in the final analysis (**Fig 1**). The detailed morbidity of pathologies is described in the supplementary Table 2 (see **S2 Table**). **Table 1** summarizes socio-demographic and life style characteristics of patients according to pathology groups. Two-thirds were female, although men are overrepresented among HIV and hematology groups. Regarding clinical data, almost a quarter of participants declared occasional or frequent alcohol consumption. Respectively, 37% and 31% of participants declared abdominal pain and diarrhoea during the last month (see **Table 2**).

**Table 1:** Socio-demographic and life style characteristics of patients according to pathology groups.

Characteristics	All patients n = 1,151	HIV n = 344	Rheumatology n = 334	Oncology n = 371	Hematology n = 102
	n (%)	n (%)	n (%)	n (%)	n (%)
**Age** (mean, SD)	46.4 (14.7)	38.1 (12.1)	50.4 (15.0)	50.4 (13.0)	46.6 (16.2)
**Gender**					
*male*	377 (32.8)	220 (64.0)	42 (12.6)	60 (16.2)	55 (53.9)
*female*	774 (67.2)	124 (36.0)	292 (87.4)	311 (83.8)	47 (46.1)
**Education level**					
*Primary school*	400 (34.8)	116 (33.7)	77 (23.1)	171 (46.1)	36 (35.3)
*Secondary school*	453 (39.3)	140 (40.7)	141 (42.2)	138 (37.2)	34 (33.3)
*Technical school*	64 (5.6)	26 (7.6)	23 (6.9)	14 (3.8)	1 (1.0)
*University*	234 (20.3)	62 (18.0)	93 (27.8)	48 (12.9)	31 (30.4)
**Living area**^**a**^					
*Rural*	172 (15.0)	59 (17.2)	41 (12.3)	57 (15.4)	15 (15.0)
*Urban*	976 (85.0)	285 (82.8)	293 (87.7)	313 (84.6)	85 (85.0)
**Region**					
*Cochabamba*	573 (49.8)	168 (48.8)	163 (48.8)	169 (45.6%)	73 (71.6)
*Santa Cruz*	578 (50.2)	176 (51.2)	171 (51.2)	202 (54.4)	29 (28.4)
**Walks without shoes when in tropical area**^**a**^					
*Never*	306 (26.6)	97 (29.4)	102 (33.1)	72 (20.5)	35 (36.1)
*Sometimes/frequently*	781 (73.4)	233 (70.6)	206 (66.9)	280 (79.5)	62 (63.9)

^a^Missing data: living area (n = 3), walking without shoes when in tropical or subtropical area (n = 64:participants from Cochabamba who never lived or travelled to tropical or subtropical area)

SD = standard deviation

**Table 2:** Clinical characteristics and immunosuppressive treatment of patients according to pathology groups.

	All patients n = 1151	HIV n = 344	Rheumatology n = 334	Oncology n = 371	Hematology n = 102
	n (%)	n (%)	n (%)	n (%)	n (%)
**Alcohol consumption**					
*Never*	890 (77.3)	212 (61.6)	293 (87.7)	300 (80.9)	85 (83.3)
*Occasional/frequently*	261 (22.6)	128 (38.4)	40 (12.3)	71 (19.1)	17 (16.7)
**Corticoids (last 3 months)**					
*Yes*	243 (21.1)	1 (0.3)	204 (61.1)	23 (6.2)	15 (14.7)
*No*	908 (78.9)	343 (99.7)	130 (38.9)	348 (93.8)	87 (85.3)
**Immunosuppressive drug (other than corticoids) in last 3 months**					
*Yes*^*a*^	249 (21.6)	0 (0)	170 (50.9)	51 (13.7)	28 (27.5)
*No*	902 (78.4)	344 (100)	164 (49.1)	320 (86.3)	74 (72.5)
**Other intestinal helminth(s)**					
*Yes*^*b*^	52 (4.5)	23 (6.7)	4 (1.2)	20 (5.4)	5 (4.9)
*No*	1099 (94.5)	321 (93.3)	330 (98.8)	351 (94.6)	97 (95.1)
**Pathogenic intestinal protozoa**					
*Yes*^*c*^	109 (9.5)	44 (12.8)	28 (8.4)	26 (7.1)	11 (10.8)
*No*	1042 (89.5)	300 (87.2)	306 (91.6)	345 (92.9)	91 (89.2
**Abdominal pain during last month**					
*Yes*	425 (36.9)	134 (39.0)	83 (24.9)	174 (46.9)	34 (33.3)
*No*	726 (63.1)	210 (61.0)	251 (75.1)	197 (53.1)	68 (66.7)
**Diarrhea during last month**					
*Yes*	344 (30.8)	129 (37.5)	76 (22.8)	108 (29.1)	41 (40.2)
*No*	797 (69.2)	215 (62.5)	258 (77.2)	263 (70.9)	61 (59.8)

^a^*Rheumatology*: methotrexate (n = 115); leflunomide (n = 22); azathioprine (n = 20); leflunomide + methotrexate (n = 8); azathoprine + methotrexate (n = 1); leflunomide + rituximab (n = 1); mycophenolate (n = 1); cyclophosphamide (n = 1); adalimumab (n = 1); *Oncology*: chemotherapy (n = 51); *Hematology*: chemotherapy (n = 28)

^b^*Ascaris lumbricoides*: 1.6% (n = 18/1151), *Hymenolepis nana* 1.3% (n = 15/1151), *Ancylostoma sp* 0.9% (n = 10/1151), *Trichuris trichiuria* 0.5% (n = 6/1151), *Enterobius vermicularis*: 0.3% (n = 4/1151)

^c^*Giardia lamblia*: 4.4% (n = 44/1151), *Entamoeba histolytica/dispar*: 3.7% (n = 43/1151), *Cryptosporidium sp*: 1.5% (n = 17/1151), *Isospora belli*: 0.4% (n = 5/1151).

### Prevalence of *Strongyloides stercoralis*

The observed serological and coproparasitological prevalence was 23.0% (95% CI, 20.7–25.5; n = 265/1,151) and 7.6% (95% CI, 6.2–9.3; n = 88/1,151), respectively. Based on the sensitivity and the specificity of ELISA tests, the estimation of the actual prevalence of *S*. *stercoralis* was 20.2% (95% CI, 17.9–22.5). **S3 Table** details coproparasitological and serological prevalence according to pathology group and region (See **S3 Table**).

### Prevalence of other parasites

Five helminths other than *S*. *stercoralis*, and four pathogenic protozoa were detected in the stools of participants (prevalence ranging between 0.3% and 4.4%, see **Table 2**).

### Factors associated with *S*. *stercoralis*

**Table 3** summarizes the factors associated with *S*. *stercoralis*, as determined by bivariate analysis. Low education level, younger age and barefoot walking were significantly associated with positive coproparasitology and positive serology. Male gender and living in rural areas were associated with the presence of stool larvae, but not with positive serology. Living in Cochabamba rural, compared to urban areas, was associated with stool larvae (16/87; 18.4% *vs*. 35/483; 7.2%; p<0.001) and with positive serology (29/87; 33.3% *vs*. 96/483; 19.9%; p = 0.001). Among participants in Santa Cruz, living in rural area was not associated with stool larvae (6/85; 7.1% *vs*. 31/493; 6.3%; p = 0.79), nor with positive serology (19/85; 22.4% *vs*. 120/493; 24.3%; p = 0.69).

**Table 3:** Socio-demographic, life style and clinical factors associated with *S*. *stercoralis* in bivariate analysis, according to coproparasitological and serological results.

Characteristics		Positive coproparasitology (n = 88)			Positive serology (n = 265)		
	n	n (%)	OR (95% CI)	p	n (%)	OR (95% CI)	p
**Region**							
*Cochabamba*	573	51 (8.9)	1.4 (0.9–2.2)	0.11	126 (22.0)	0.9 (0.7–1.2)	0.40
*Santa Cruz*	578	37 (6.4)	(ref)		139 (24.0)	(ref)	
**Age**							
*<40 years*	384	42 (10.9)	**3.9 (1.8–9.4)**	**0.002**	99 (25.8)	**1.7 (1.1–2.6)**	**0.047**
*≥40-<60 years*	540	39 (7.2)	**2.4 (1.1–6.0)**		127 (23.5)	**1.5 (1.0–2.2)**	
*≥60 years*	227	7 (3.1)	(ref)		39 (17.2)	(ref)	
**Gender**							
*Men*	377	40 (10.6)	**1.8 (1.2–2.8)**	**0.008**	89 (23.6)	1.1 (0.8–1.4)	0.74
*Women*	774	48 (6.2)	(ref)		176 (22.7)	(ref)	
**Education level**							
*Primary school*	400	55 (13.8)	**9.1 (3.6–30.1)**	**<0.001**	126 (31.5)	**2.7 (1.8–4.2)**	**<0.001**
*Secondary school*	517	29 (5.6)	**3.4 (1.3–11.5)**		105 (20.3)	1.5 (1.0–2.3)	
*Technical/ University*	234	4 (1.7)	(ref)		34 (14.5)	(ref)	
**Living area**							
*Rural*	172	22 (12.8)	**2.0 (1.2–3.3)**	**0.006**	48 (27.9)	1.4 (0.9–2.0)	0.097
*Urban*	976	66 (6.8)	(ref)		216 (22.1)	(ref)	
**Walks without shoes** **in tropical area**							
*Sometimes/frequently*	781	68 (8.7)	**1.8 (1.1–3.4)**	**0.034**	195 (25.0)	**1.4 (1.0–1.9)**	**0.046**
*Never*	306	15 (4.9)	(ref)		59 (19.3)	(ref)	
**Alcohol consumption**							
*Occasionally/frequently*	261	29 (11.1)	**1.9 (1.1–3.0)**	**0.009**	63 (24.1)	1.1 (0.8–1.5)	0.63
*Never*	889	56 (6.3)	(ref)		202 (22.7)	(ref)	
**Corticoids use (last three months)**							
*Yes*	243	10 (4.1)	(ref)	**0.02**	52 (21.4)	(ref)	0.50
*No*	908	78 (8.6)	**2.2 (1.2–4.5)**		213 (23.5)	1.1 (0.8–1.6)	
**Immunosuppressive drug other than corticoids (last 3 months)**							
*Yes*^*a*^	249	15 (6.0)	(ref)	0.28	52 (20.9)	(ref)	0.36
*No*	902	73 (8.1)	1.4 (0.8–2.5)		213 (23.5)	1.2 (0.8–1.7)	
CD4^+^ count (cells/ml)							
HIV<300	171	31 (18.1)	**3.2 (2.0–5.1)**	**<0.001**	43 (25.2)	1.0 (0.7–1.4)	0.98
HIV≥300 or HIV not known	880	57 (6.5)	(ref)		222 (25.2)	(ref)	
**Other intestinal helminths**							
*Yes*^*a*^	52	11 (21.2)	**3.6 (1.7–7.1)**	**<0.001**	17 (32.7)	1.7 (0.9–3.0	0.09
*No*	1099	77 (7.0)	(ref)		248 (22.6)	(ref)	
**Pathogenic intestinal protozoa**							
*Yes*^*a*^	109	10 (9.2)	1.2 (0.6–2.4)	0.55	19 (17.4)	0.7 (0.4–1.2)	0.14
*No*	1042	78 (7.5)	(ref)		246 (23.6)	(ref)	
**Diarrhea during last month**							
*Yes*	354	38 (10.7)	**1.8 (1.1–2.8)**	**0.009**	87 (24.6)	1.1 (0.8–1.5)	0.4
*No*	797	50 (6.3)	(ref)		178 (22.3)	(ref)	
**Abdominal pain during last month**							
*Yes*	425	45 (10.6)	**1.9 (1.2–2.9)**	**0.004**	108 (25.4)	1.2 (0.9–1.6)	0.14
*No*	726	43 (5.9)	(ref)		157 (21.6)	(ref)	

^a^See **S2 Table**

There was no difference in prevalence between Cochabamba and Santa Cruz as defined by coproparasitology (6.4% *vs*. 8.9%; p = 0.11) or serology (24.0% *vs*. 22.0%; p = 0.4). Among 64 patients in Cochabamba who had never travelled to the tropical lowlands, five (7.8%) had a positive coproparasitology.

Among clinical characteristics, being HIV-positive with fewer than 300 CD4^+^ cells/ml, declaring occasional or frequent alcohol consumption, and abdominal pain and diarrhea in the last month were factors significantly associated with stool larvae. No association was found between these factors and serological positivity.

**Table 4** summarizes socio-demographic and potential exposure factors associated with *S*. *stercoralis* from multivariate analysis. Younger age, lower education levels, living in Cochabamba, walking barefoot, drinking alcohol and being HIV-positive with low CD4^+^ counts were associated with finding stool larvae. Only younger age and lower education level were associated with a positive serology.

**Table 4:** Socio-demographic and life style characteristics associated with *S*. *stercoralis* in multivariate analysis, according to coproparasitological and serological results.

	Positive coproparasitology		Positive serology	
	Adjusted OR (95% CI)	p	Adjusted OR (95% CI)	p
*Cochabamba vs*. Santa Cruz*	2.0 (1.2–3.4)	**0.01**	1.0 (0.7–1.5)	0.90
*Age 10 years difference*	0.7 (0.6–0.8)	**<0.001**	0.8 (0.8–0.9)	**0.001**
*Men vs*. women*	1.5 (0.9–2.6)	0.11	1.1 (0.8–1.6)	0.44
*Primary school* *Secondary school /technical* *University*	13.6 (4.6–39.8) 3.5 (1.2–10.3) -	**<0.001** **0.024** (ref)	3.1 (2.0–4.9) 1.7 (1.1–2.6) -	**<0.001** **0.02** (ref)
*Rural vs*. urban*	1.6 (0.9–2.7)	0.12	1.2 (0.8–1.8)	0.27
*Barefoot sometimes or frequently* *	2.6 (1.3–5.0)	**0.005**	1.4 (0.9–2.0)	0.11
OH consumption occasional or frequently*	1.7 (1.0–2.9)	**0.043**	1.0 (0.7–1.4)	0.97
Corticoids during last three months*	1.1 (0.5–2.4)	0.76	1.2 (0.8–1.8)	0.31
*CD4*^*+*^ counts <300 /ml*	2.1 (1.2–3.8)	**0.01**	0.9 (0.6–1.4)	0.74

*reference group indicated in Table 3

OR: odds ratio; CI: confidence interval

Performance of the different coproparasitological diagnostic methods is presented in Table 5. Among 37 stool samples with detection of larvae with DSE, 3 samples were negative with APC and MBT. Among 39 stool samples with detection of larvae with FGST, 2 samples were negative with APC and MBT. DSE and FGCT, respectively, identified 4.2% (3/72) and 2.8% (2/72) additional positive cases (not identified with APC and MBT).

**Table 5:** *S*. *stercoralis* larvae in direct smear examination, formalin-gasoline sedimentation technique, agar plate culture and modified Baermann technique of two stool samples*.

	DSE		FGST		APC		MBT		Combined 4 methods	
	n	%	n	%	n	%	n	%	n	%
Cumulative result after examination of										
1 stool sample	20	2.5	17	2.1	48	6.0	46	5.7	53	6.6
2 stool samples	29	3.6	31	3.9	61	7.6	64	8.0	72	9.0
Sensitivity** (2 samples)	29/72	40.3	31/72	43.1	61/72	84.7	64/72	88.9		
Sensitivity** of an individual test	37/144	25.7	39/144	27.1	97/144	67.4	100/144	69.4		

*stool results processed in CENETROP laboratory were not included (see methods section)

**sensitivity among people with *S*. *stercoralis* larvae detected with at least one coproparasitological technique (the global sensitivity of each test is not computable due to the lack of gold standard for the diagnosis of *S*. *stercoralis*)

DSE = direct smear examination, FGST = formalin-gasoline sedimentation technique, APC = agar plate culture, MBT = modified Baermann technique

## Discussion

### High prevalence of *S*. *stercoralis* among patients at high risk of complications

This study demonstrated that strongyloidiasis is widely present in Bolivia, with an estimated actual prevalence of 20% among patients at high risk of complications in the Cochabamba and Santa Cruz departments. Until now, strongyloidiasis was largely ignored in Bolivia, due to the lack of both epidemiological data and availability of sensitive diagnostic tools. The rare studies that previously quantified the prevalence of strongyloidiasis (ranging from 0.9% to 5.1%) were performed in a small number of communities, different population and used insensitive coproparasitologic techniques [17–19]. In villages of the Chaco Department, in rural areas of southern Bolivia, serological prevalence of 16% and 6% were reported in 1987 and 2013, respectively [20]. Nonetheless our results are consistent with a serological survey reporting strongyloidiasis among 16% of migrants native from Bolivia in Italy [21]. Bolivia is not an exception in under-reporting strongyloidiasis due to poor sensitivity of most fecal based methods. In most of African, Asian, and Latin American endemic countries, reports of severe and fatal strongyloidiasis are exceedingly rare, which suggests that most cases are simply missed [2, 3, 5]; in contrast, cases of severe strongyloidiasis are reported among migrants in industrialized (non-endemic) countries. In Europe, cases of hyperinfection syndrome and disseminated strongyloidiasis were reported in two patients originating from Bolivia [22, 23].

### Hyperendemicity of *S*. *stercoralis* in a temperate-climate inter-Andean valley

Strongyloidiasis, which is endemic in tropical and subtropical regions, was believed to occur only sporadically in temperate areas [8, 24, 25]. This study finds however equally high prevalence in the tropical region of Santa Cruz and the temperate region of Cochabamba, at least in patients at high-risk of complications. A substantial (7.8%) proportion of patients in Cochabamba who had never travelled to the tropical lowlands had a positive fecal result, a highly reliable test (specificity of 100%). To our knowledge, this is the first study that reports that *S*. *stercoralis* is transmitted in a temperate region, at an elevation over 2,500 meters.

### Factors associated with *S*. *stercoralis* infection

*Low level of education* is a factor highly associated with strongyloidiasis infection in our study population in agreement with previous reports [26].

*Younger age* is also associated with infection, with a 20–30% decreasing prevalence per decade of life, according to our multivariate analysis. This result was surprising, as earlier studies in South America, Asia and Africa linked infection with increasing age, due to the maintenance of *S*. *stercoralis* in the human body for decades (by auto-infection) [5, 26–29]. Alternatively in Bolivia, where increase in temperature and precipitation was recorded during the past 30 years, including in the inter-Andean valleys [30], ongoing climate change may favour the ecology and extend the geographical range of the free life-form of the parasite. The observed prevalence in young patients may therefore reflect a recent change in parasite transmission dynamics. Another hypothesis could explain the negative association between age and *S*. *stercoralis* infection. It is possible that older people with *S*. *stercoralis* infection have a higher mortality than those without an infection. Older infected people may be consequently underrepresented due to excess mortality.

Infection was also more common among people declaring *walking barefoot*. This risk factor, correlated with age, rural residence and low education level, is regularly reported in the literature [4, 29]. No gender difference was observed on multivariate analysis, similarly to some previous studies [26, 31]. The association of being a man and a positive fecal test in the bivariate analysis is likely to be confounded by other predictive factors (e.g. HIV infection).

The presence of stool larvae was associated with *alcohol consumption* and *lower CD4*^+^ *counts* in bivariate and multivariate analysis. These factors were however not associated with positive serology. Pathophysiological mechanisms could explain these observations. For example, excessive alcohol consumption increases the levels of endogenous corticosteroids, mimicking the effect of helminthic ecdysteroid hormones and increasing the fecundity in females and the maturation of larvae [32–34]. Advanced HIV disease and low CD4^+^ counts are associated with a higher number of intestinal non-invasive larvae in the stools [35, 36]. Alcohol consumption and advanced HIV thus lead to higher intestinal parasite loads, thereby facilitating their detection in the stool, without an increased infection rate.

Clinical symptoms like *diarrhea* and *abdominal pain* were associated with the presence of larvae, but not with positive serology. This is possibly explained by a better sensitivity of coproparasitological tests at high parasitic loads. While chronic *S*. *stercoralis* infection is often asymptomatic, diarrhea and abdominal pain are more common in patients with high intestinal parasite loads [37].

In resource-poor countries, strongyloidiasis is often less prevalent in *urban* than in *rural* areas, related to ecological and sanitation conditions [38–40]. While the overall multivariate model revealed a non-significant trend between areas and infection in this study, a strong association was found only for Cochabamba. Such differences can be explained by the extent of paved/unpaved roads and mud being deposed during the rainy season, and whether people wear closed shoes or go barefoot. Indeed, walking with open shoes in the mud is more common in urban Santa Cruz than in Cochabamba during the rainy season, due to differences in infrastructure, climate and habits of the population.

### Coproparasitological and serological diagnostic methods

Performance comparison of coproparasitological techniques confirms that APC and MBT are much more sensitive than DSE and FGST [41]. DSE and FGST contribute to few additional positive cases detected when they are performed concomitantly with MBT and APT. Five stool samples with positive DSE or SGST had negative MBT and APC. These cases likely correspond to participants who did not follow the instructions for collection and delivery of stool; the delay between stool emission and analysis was probably too long (dead larvae). This data confirms that these methods are effective, as far as the preanalytical requirements are respected, to guarantee their realization on fresh stools. In any case, efforts must be strengthened to implement and to facilitate access to serological and effective coproparasitological diagnostic techniques in Bolivia. The Baermann technique should be preferred over the agar plate culture, because this method is faster, safer, and more affordable with comparable sensitivity [4, 35].

### Strengths and weaknesses

To our knowledge, this study is the largest investigating the epidemiology of strongyloidiaisis among patients immunosuppressed at high risk of complications. Another strength is the use of multiple laboratory tests for the diagnosis of this disease for which no gold standard exists: in addition to serology test, two stool samples were analyzed by four methods. A first limitation relates to the participation rate of 62%, with 34.6% of patients having difficulty collecting stools or refusing the blood test, despite lab tests being free of charge for the study participants. Patients who refused to participate or who did not provide all stool and/or blood samples for laboratory analysis were more likely to live in remote rural areas and were more likely to have lower education levels; because a low level of education is associated with the infection, we think that the reported *S*. *stercoralis* prevalence could be underestimated. Second, the accuracy of serology is high, but false-positive and false-negative results can occur [16]. To correct these inaccuracies, we estimate the actual prevalence, which takes into account the sensitivity and specificity of the serological test.

### Public health and therapeutic implications

In the study population, abdominal pain and diarrhea are symptoms associated with *S*. *stercoralis* infection. These symptomatic patients must be diagnosed and treated, because Forrer et al have recently demonstrated that these symptoms decline significantly after ivermectin administration [42]. Nevertheless, strategies must take into account asymptomatic patients, because most chronically infected people do not have symptoms. In groups of patients at high risk of complications and with a >10% prevalence of *S*. *stercoralis*, experts recommend empiric treatment with ivermectin, after considering the limited performance and relative sophistication of current diagnostic tools, the difficulty to collect stools (as observed in our study), the life-threatening nature of strongyloidiasis and the high drug safety [16]. Given the high prevalence found in our study and the sparsity of diagnostic facilities in the country, this approach should be strongly encouraged in Bolivia, in both tropical and temperate regions [43]. Moreover this recommendation should not neglect patients with low level of education, as this factor is highly associated with strongyloidiasis infection in the study population. We strongly recommend that all patients should be dewormed when suffering from a disease that either induces immunosuppression, or potentially triggers the intake of immunosuppressive drugs.

If such levels of prevalence are found in the general population, then adding ivermectin to the existing deworming programs should be discussed. The national deworming campaigns of the Bolivian Ministry of Health currently consist of the prescription of a 400 mg dose of albendazole in primary schools, which is of suboptimal efficacy for *S*. *stercoralis* [6].

## Conclusions

The study demonstrates that strongyloidiasis is widespread in Bolivia among patients at high risk of complications. *S*. *stercoralis* is not limited to tropical and subtropical regions in Bolivia, but also appears to be endemic in the Andean valleys above 2,500 meters.

The authors recommend that Bolivia reinforces control strategies to prevent complications from this serious parasitic disease. Because *Strongyloides stercoralis* is the most dangerous enteric parasite, laboratory methods sensitive for *S*. *stercoralis* must be accessible to monitor prevalence and treatment. Given the high prevalence in this population, the mortality associated with HS/DS and the efficacy and safety of early treatment, we recommend the presumptive use of ivermectin for patients that undergo immunosuppression, or before initiating immunosuppressive therapy. Another recommendation is the introduction of preventative measures such as promoting wearing shoes.

## Supporting information

S1 Dataset (XLSX)

S1 Table Strobe statement. (DOC)

S2 Table Pathologies affecting patients included in the study. (DOCX)

S3 Table Coproparasitological and serological prevalence of strongyloidiasis according to pathology group. (DOCX)
