# Supplementary material for: Epidemiology of Strongyloides stercoralis infection in Bolivian patients at high risk of complications
Source: PLoS Negl Trop Dis. 2019 Jan 17;13(1):e0007028. doi: 10.1371/journal.pntd.0007028 (PMC6353209; doi:10.1371/journal.pntd.0007028)
Supplement: S1 Table — (DOC) [file pntd.0007028.s002.doc]

**S1 Table. STROBE Statement**—Checklist of items that should be included in reports of cross-sectional studies

**“Epidemiology of Strongyloides stercoralis infection in Bolivian patients at high risk of complications”**

|  | Item No | Recommendation | Section / paragraph | Relevant text from manuscript |
| --- | --- | --- | --- | --- |
| **Title and abstract** | 1 | (*a*) Indicate the study’s design with a commonly used term in the title or the abstract | Abstract | Abstract: A cross-sectional multi-center prevalence study |
|  |  | (*b*) Provide in the abstract an informative and balanced summary of what was done and what was found | Abstract | Please read the abstract |
| Introduction | | |  |  |
| Background/  rationale | 2 | Explain the scientific background and rationale for the investigation being reported | Introduction  1^st^ paragraph | First paragraph: epidemiology of strongyloidiasis |
|  |  |  | 2^nd^ paragraph | Second paragraph: infection’s mode and short summary of the cycle: (information necessary to understand the severity of the disease) |
|  |  |  | 3^rd^ – 5^th^ paragraphs | Third paragraph: symptoms of strongyloidiasis, risk factors of severe strongyloidiasis and treatment |
| Objectives | 3 | State specific objectives, including any prespecified hypotheses | Last paragraph | Objectives: Please read the last paragraph of the introduction |
|  |  |  | Introduction  1st paragraph | Hypotheses: intro, first paragraph “epidemiological data are largely lacking in low- and middle-income countries such as Bolivia” => Our implied hypothesis: strongyloidiasis should be overlooked in Bolivia |
| Methods | | |  |  |
| Study design | 4 | Present key elements of study design early in the paper | Methods  1st section | Please read the ‘Study setting”’ section => cross-sectional study design |
| Setting | 5 | Describe the setting, locations, and relevant dates, including periods of recruitment, exposure, follow-up, and data collection | Methods  1st section | Locations and relevant dates – period of recruitement : read the “Study setting” section |
|  |  |  | Methods  6th section | -Follow-up: Please read the 3 last lines of the “Ethical considerations” section (clinical management of participants) |
|  |  |  | Methods  4th section | -data collection: The data were compiled into an Excel spreadsheet (See S1 dataset). Please read the “Data collection” section |
| Participants | 6 | (*a*) Give the eligibility criteria, and the sources and methods of selection of participants | Methods  2nd section | Please read the ‘Study population, inclusion/exclusion criteria and sample size”  section |
| Variables | 7 | Clearly define all outcomes, exposures, predictors, potential confounders, and effect modifiers. Give diagnostic criteria, if applicable | Methods  3^rd^ & 4^th^ sections | Please read “Data collection” and “statistical analysis” sections and “Table 2” for socio-demographic and clinical variables, and “Laboratory investigation procedures” for the diagnostics criteria of strongyloidiasis infection |
| Data sources/ measurement | 8* | For each variable of interest, give sources of data and details of methods of assessment (measurement). Describe comparability of assessment methods if there is more than one group | Methods  3^rd^ -4^th^-5^th^ sections | Please read “Data collection” and “statistical analysis” and “Laboratory investigation procedures” sections. For the accuracy of the serological test, please read the first 4 lines of the “Estimation of the actual prevalence” section |
| Bias | 9 | Describe any efforts to address potential sources of bias | Methods  4th and 5th sections | 1) accuracy of serology is high, but false-positive and false-negative results can occur (and there is no gold standard for the diagnosis of *S.stercoralis*) => to limit this bias, we calculate the actual prevalence, which takes into account the sensitivity and specificity of the serological test (please read “Estimation of the actual prevalence” section)  2) We used a multivariable method to estimate the association between several factors and positive exams for strongyloidiasis, taking into account some potential confounding factors (please read “statistical analysis“ section). |
| Study size | 10 | Explain how the study size was arrived at | Methods  2^nd^ section | Please read the 6 last lines of the section ‘Study population, inclusion/exclusion criteria and sample size” section |
| Quantitative variables | 11 | Explain how quantitative variables were handled in the analyses. If applicable, describe which groupings were chosen and why |  | n/a |
| Statistical methods | 12 | (*a*) Describe all statistical methods, including those used to control for confounding | Methods  4^th^ section | Please read the section “statistical analysis” section |
|  |  | (*b*) Describe any methods used to examine subgroups and interactions |  | n/a. |
|  |  | (*c*) Explain how missing data were addressed | Methods  4^th^ section | Please read the “statistical analysis“ section |
|  |  | (*d*) If applicable, describe analytical methods taking account of sampling strategy |  | n/a |
|  |  | (*e*) Describe any sensitivity analyses |  | n/a |
| Results | | |  |  |
| Participants | 13* | (a) Report numbers of individuals at each stage of study—eg numbers potentially eligible, examined for eligibility, confirmed eligible, included in the study, completing follow-up, and analysed | Results  1^st^ section  & Fig 1 | Please read the first 4 lines af the “patient characteristics” section |
|  |  | (b) Give reasons for non-participation at each stage | Fig 1 | Please see Fig 1 |
|  |  | (c) Consider use of a flow diagram | Fig 1 | Please see Fig 1 |
| Descriptive data | 14* | (a) Give characteristics of study participants (eg demographic, clinical, social) and information on exposures and potential confounders | Results  1^st^ section | Please read “patient characteristics” section; S1 Table & Table 1 |
|  |  | (b) Indicate number of participants with missing data for each variable of interest | Results  1^st^ section | Please see footnotes of Table 1 and S2 Table |
| Outcome data | 15* | Report numbers of outcome events or summary measures | Results  2^nd^ section &  S4 Table | Please see section “Prevalence of Strongyloides stercoralis” and S4 Table |
| Main results | 16 | (*a*) Give unadjusted estimates and, if applicable, confounder-adjusted estimates and their precision (eg, 95% confidence interval). Make clear which confounders were adjusted for and why they were included | Results  2^nd^ section  Table 2 & 3 | Please read Factors associated with S. stercoralis” section. For inclusion of potential confounders: see “statistical analysis” section (methods) |
|  |  | (*b*) Report category boundaries when continuous variables were categorized |  | n/a |
|  |  | (*c*) If relevant, consider translating estimates of relative risk into absolute risk for a meaningful time period |  | n/a |
| Other analyses | 17 | Report other analyses done—eg analyses of subgroups and interactions, and sensitivity analyses |  | n/a |
| Discussion | | |  |  |
| Key results | 18 | Summarise key results with reference to study objectives | Discussion  Sections 1-4 | Please read the discussion, sections 1 to 4 |
| Limitations | 19 | Discuss limitations of the study, taking into account sources of potential bias or imprecision. Discuss both direction and magnitude of any potential bias | Discussion  6th section | Please read section “strengths and weaknesses” |
| Interpretation | 20 | Give a cautious overall interpretation of results considering objectives, limitations, multiplicity of analyses, results from similar studies, and other relevant evidence | Discussion  6^th^ section  Conclusion | Overall interpretation of results: please read “Public health and therapeutic implications” section and the conclusion |
|  |  |  | Discussion | Comparison with the results of other studies - prevalence: read 13 last lines 1^st^ section; factors associated: read 4^th^ section |
| Generali-sability | 21 | Discuss the generalisability (external validity) of the study results | Discussion | N/A |
| Other information | | |  |  |
| Funding | 22 | Give the source of funding and the role of the funders for the present study and, if applicable, for the original study on which the present article is based |  | information transmitted during the submission process |
