# Supplementary material for: Epidemiology of Strongyloides stercoralis infection in Bolivian patients at high risk of complications
Source: PLoS Negl Trop Dis. 2019 Jan 17;13(1):e0007028. doi: 10.1371/journal.pntd.0007028 (PMC6353209; doi:10.1371/journal.pntd.0007028)
Supplement: S2 Table — (DOCX) [file pntd.0007028.s003.docx]

**S2 Table: Pathologies affecting patients included in the study**

| HIV (344 patients) | Rheumatic disease (334 patients) |
| --- | --- |
| CD 4 value (cells/ml)  1) 0-199: 32.3% ( n=111))  2) 200-299 : 17.4% (n=60)  3) 300-499: 33.4% (n=115)  4) ≥500: 16.9% (n=58) | **Type of rheumatic disease**  1) Autoimmune rheumatic diseases^a^: 78.4% (n=262)  2) Other rheumatic diseases^b^: 21.6% (n=72) |
| Oncological disease (371 patients) | **Hematologic disease (102 patients)** |
| Sites of primary oncological disease  1) Female reproductive organs: 41.8% (n=155)  2) Breast: 30.0% (n=111)  3) Digestive system: 11.0% (n=41)  4) Prostate: 3.5% (n=13)  5) Head and neck : 3.5% (n=13)  6) Other: 10.2% ( n=38)^c^ | **Type of hematologic disease**  1) Non-Hodgkin lymphoma: 28.4% (n=29)  2) Chronic myelogenous leukemia: 24.5% (n=25)  3) Acute lymphocytic leukemia: 14.7% (n=15)  4) Hodgkin lymphoma: 10.8% (n=11)  5) Acute myelogenous leukemia: 4.9% (n=5)  6) Other:16.7% (n=17)^d^ |

^a^Autoimmune rheumatic diseases: rheumatoid arthritis (n=207); systemic lupus erythematosus (n=38); scleroderma (n=5); Sjoegren’s syndrome (n=5); Wegener’s granulomatosis (n=2); undetermined vasculitis (n=2); polymyositis (n=1); mixed connective tissue disease (n=1); psoriatic arthritis (n=1).

^b^Other rheumatic diseases that can indicate transiently steroid prescriptions: erosive degenerative osteoarthritis (n=35); other degenerative joint disease (n=32); chronic tendinitis (n=5)

^c^Other oncological primary disease sites: skin (n=12); bones or soft tissues (n=9) ; lungs (n=7) ; central nervous system (n=6) ; urinary tract (n=4)

^d^Other hematologic diseases : multiple myeloma (n=5); chronic lymphocytic leukemia (n=2); myelodysplasic syndrom (n=2); myeloproliferative disorder (n=2); hemolytic anemia (n=2) ; [idiopathic thrombocytopenic purpura](http://www.mayoclinic.org/diseases-conditions/idiopathic-thrombocytopenic-purpura/basics/definition/con-20034239) (1) ; thymoma (n=1) ; indeterminate (n=2)
