# Supplementary material for: Epidemiology of Strongyloides stercoralis infection in Bolivian patients at high risk of complications
Source: PLoS Negl Trop Dis. 2019 Jan 17;13(1):e0007028. doi: 10.1371/journal.pntd.0007028 (PMC6353209; doi:10.1371/journal.pntd.0007028)
Supplement: S3 Table — (DOCX) [file pntd.0007028.s004.docx]

**S3 Table: Coproparasitological and serological prevalence of strongyloidiasis according to pathology group**

|  | Positive coproparasitology | | | Positive serology | | | Actual prevalence |
| --- | --- | --- | --- | --- | --- | --- | --- |
|  | Cochabamba | Santa Cruz | Total | Cochabamba | Santa Cruz | Total | Total |
|  | n  (%) | n  (%) | n  (%) | n  (%) | n  (%) | n  (%) | %  (95% CI) |
| ALL PATIENTS | **51/573**  **(8.9)** | **37/578**  **(6.4)** | **88/1151**  **(7.6)** | **126/573**  **(22.0)** | **139/578**  **(24.0)** | **265/1151**  **(23.0)** | **20.2**  **(17.9-22.5)** |
| HIV | 29/168  (17.3) | 12/176  (6.8) | 41/344  (11.9) | 44/168  (26.2) | 42/176  (23.9) | 86/344  (25.0) | 22.4  (12.0-32.8) |
| Rheumatology | 4/163  (2.5) | 4/171  (2.3) | 8/334  (2.4) | 30/163  (18.4) | 34/171  (19.9) | 64/334  (19.2) | 15.5  (6.0-25.6) |
| Oncology | 10/169  (5.9) | 18/202  (8.9) | 28/371  (7.5) | 41/169  (24.3) | 57/202  (28.2) | 98/371  (26.4) | 24.0  (17.7-35.1) |
| Hematology | 8/73  (11.0) | 3/29  (10.3) | 11/102  (10.8) | 11/73  (15.1) | 6/29  (20.7) | 17/102  (16.7) | 12.6  (0.0-30.7) |
